# PPI in preventing gastrointestinal injury in minor ischemic stroke or TIA patients at low risk of gastrointestinal bleeding treated with short-term dual antiplatelet therapy

**DOI:** 10.3389/fphar.2025.1644148

**Published:** 2025-07-30

**Authors:** Lu Cao, Kejia Quan, Dan Zhang, Rui Li, Nan Zhou, Peng Zhang

**Affiliations:** ^1^ Department of Pharmacy, Shaanxi Provincial People’s Hospital, Xi’an, Shaanxi, China; ^2^ Department of Pharmacy, The First Hospital of Weinan City, Weinan, Shaanxi, China; ^3^ Department of Neurology, Shaanxi Provincial People’s Hospital, Xi’an, Shaanxi, China

**Keywords:** minor IS/TIA, dual antiplatelet therapy, PPI, gastrointestinal injury, pneumonia

## Abstract

**Objective:**

To analyse the use of prophylactic proton pump inhibitors (PPIs) and their benefits for the prevention of gastrointestinal injury and to determine the optimal course of preventive use among patients with minor ischaemic stroke (IS) and transient ischaemic attack (TIA) at low risk of gastrointestinal bleeding (GIB) treated with short-term dual-antiplatelet therapy (DAPT).

**Methods:**

We retrospectively collected clinical data from the hospital information system (HIS) from January 2022 to December 2023. The data were collected from patients who were admitted to a tertiary hospital with a first occurrence of minor IS/TIA diagnosed within 14 days and treated with short-term DAPT. Univariate and multivariate logistic regression analyses were used to explore the correlations between the use of PPIs, different treatment durations, and the incidence rates of GIB, gastrointestinal discomfort, other types of bleeding, and pneumonia in these patients.

**Results:**

A total of 220 patients were included, with 52 in the PPI group (23.64%) and 168 in the non-PPI group (76.36%). The results showed that PPI use did not significantly reduce the incidence of GIB (P = 0.059) or other types of bleeding (P = 0.916) in patients who were treated with DAPT and were at low risk of GIB. The incidence of pneumonia in the PPI group was higher than that in the non-PPI group, but the difference was not statistically significant (42.86% vs. 23.00%, P = 0.840). However, PPI use significantly reduced the occurrence of gastrointestinal discomfort (P = 0.033, OR: 0.448; 95% CI: 0.215–0.935), with no significant difference based on treatment duration (≤7 days vs. >7 days, P = 0.520).

**Conclusion:**

Regular use of PPIs within the first 7 days of initiating DAPT in patients with minor IS/TIA at low risk of GIB significantly reduces symptoms of gastrointestinal discomfort while minimizing adverse effects due to overuse of PPIs.

## 1 Introduction

Ischaemic stroke (IS) and transient ischaemic attack (TIA) are the most common types of cerebrovascular disease. In China, approximately 83% of hospitalized patients with cerebrovascular diseases suffer from IS (Chinese Medical Association Neurology Branch, 2022), which is characterized by high incidence, prevalence, recurrence, disability, and mortality rates. Studies have reported annual recurrence rates of approximately 9.6%–17.7% (Pan et al., 2021), and the rate of death or disability at 1 year poststroke is between 33.4% and 33.8% (Chinese Medical Association and Journal of Chinese Medical Association, 2021). Antiplatelet therapy, including traditional drugs such as aspirin and clopidogrel, as well as some relatively newer antiplatelet drugs such as ticagrelor, cangrelor, prasugrel, indobufen, cilostazol and eptifibatide, etc. is an effective treatment and preventative measure for IS/TIA, with guidelines recommending that patients at high risk of stroke recurrence receive combined aspirin and clopidogrel treatment within 24 h of acute noncardioembolic TIA or minor IS (National Institutes of Health Stroke Scale (NIHSS) score ≤3) for 21 days. For patients with non-cardioembolic minor IS (NIHSS score ≤5) or TIA at high risk of recurrence within 24 h of onset, accompanied by ipsilateral intracranial arterial stenosis of more than mild degree (stenosis rate >30%), aspirin combined with ticagrelor is recommended for 30 days. For patients with symptomatic severe intracranial arterial stenosis (70%–99% narrowing) within 30 days, similar treatment is recommended for 90 days after onset, followed by long-term secondary prevention with aspirin or clopidogrel alone (Chinese Medical Association Neurology Branch, 2022; Expert Committee on the Prevention and Treatment Guidelines for Thrombotic Diseases in China Chinese, 2018).

Dual-antiplatelet therapy (DAPT), which primarily consists of aspirin combined with a P2Y12 receptor inhibitor (such as clopidogrel or ticagrelor), plays a crucial role in antithrombotic treatment. However, DAPT may lead to gastrointestinal bleeding (GIB), ulcers, and other complications due to the inhibition of cyclooxygenase (COX) activity and direct damage to the gastrointestinal mucosa. The “POINT” (Platelet-Oriented Inhibition in New TIA and Minor Ischemic Stroke) trial compared DAPT with aspirin plus clopidogrel versus aspirin alone to determine if it could more effectively reduce the risk of recurrent IS within 90 days in patients with TIA or minor IS, while clarifying its safety profile (primarily bleeding risk). The results of the study showed that patients receiving DAPT had a significantly increased risk of major non-intracranial bleeding, suggesting an elevated risk of upper gastrointestinal bleeding as well (Johnston et al., 2018). Studies have suggested that approximately 2.3%–7% of patients who undergo DAPT might experience GIB (Wang, 2023), with the high-risk period occurring within 3 months of starting DAPT (Wang and Zhang, 2013). Proton pump inhibitors (PPIs), including first-generation drugs such as omeprazole, lansoprazole and pantoprazole, as well as second-generation drugs, i.e., relatively newer PPIs, such as rabeprazole and esomeprazole, which are potent inhibitors of gastric acid secretion, have been widely used to prevent gastrointestinal injuries associated with nonsteroidal anti-inflammatory drugs (NSAIDs) and antiplatelet medications (National Health Commission of the People’s Republic of China, 2021).

Currently, most evidence indicates that in patients with acute coronary syndrome (ACS) or those who have undergone percutaneous coronary intervention (PCI), the long-term use of DAPT (for at least 12 months) combined with PPIs can significantly reduce the incidence of gastrointestinal injuries, especially in patients at high risk of GIB (Wang et al., 2022; Sehested et al., 2019; Valgimigli et al., 2017). It is recommended to use PPIs for 1–3 months in addition to DAPT; for patients with a history of GIB or those who experience bleeding during antiplatelet therapy, the combined use of PPIs for 3–6 months is advised (Cardiology Discovery, 2024). However, there is little evidence regarding the benefits of PPIs in terms of preventing gastrointestinal injuries among patients with IS/TIA who are receiving short-term DAPT, and there is still no consensus regarding the guidelines. The 2023 UK and Ireland National Clinical Guidelines for Stroke recommend considering the use of PPIs alongside DAPT to reduce the risk of GIB in IS/TIA patients (National Clinical Guideline for Stroke, 2023); the 2022 U.S. Guidelines lean towards a conservative approach, recommending the combination of PPIs with DAPT only in patients with high bleeding risk (Saven et al., 2022). A retrospective cohort study published in 2025, which was based on Korea’s nationwide claims database, included 96,722 patients who were admitted for IS between 2014 and 2018, were at low risk of upper GIB, and received DAPT. Among them, 16,084 patients were treated with PPIs. The study results revealed that the use of PPIs in this patient population was associated with an average 37% reduction in the risk of significant upper GIB. However, owing to the limited use of PPIs in this patient group, more studies are needed to confirm this finding (Baik et al., 2025). Currently, there are no guidelines or studies specifically recommending treatment durations for PPI use in patients receiving short-term DAPT.

Our study aimed to enrol patients who were initially diagnosed with minor IS/TIA, at low risk of GIB and treated with DAPT for 21–90 days. By collecting relevant clinical data during hospitalization and following up at 1 year postdischarge, this study aimed to clarify the use of prophylactic PPIs and their benefits for the prevention of gastrointestinal injury in these patients. Furthermore, this study explored the appropriate duration of PPI use, thus providing evidence-based guidance for the clinical use of PPIs in such cases.

## 2 Materials and methods

### 2.1 Study design and eligibility criteria

This was a single-centre retrospective cohort study. The inclusion criteria for patients were as follows: (1) admission to our hospital from January 2022 to December 2023, age 18 years or older, diagnosis with minor acute ischaemic stroke (NIHSS ≤5) or TIA within 14 days of onset, and at low risk of GIB; and (2) DAPT during and after hospitalization, including 100 mg of aspirin once daily combined with 75 mg of clopidogrel once daily, a loading dose of ticagrelor 180 mg followed by 90 mg twice daily, or 100 mg of cilostazol twice daily, with a total treatment duration ranging from 21 to 90 days. The exclusion criteria were as follows: (1) recent severe trauma or surgery; (2) concurrent anticoagulant therapy (excluding oral anticoagulant therapy started or continued after the event but including oral anticoagulant therapy starting before the event that was switched to antiplatelet therapy afterwards); (3) concurrent bleeding disorders or coagulopathy during hospitalization; (4) active peptic ulcers or history of recurrent ulcers/bleeding within 3 months prior to admission; (5) planned use of PPIs to treat acid-related disorders (such as gastroesophageal reflux disease, GIB, or persistent gastrointestinal discomfort) or use of PPIs within the last month; (6) discordant initiation of PPI use and DAPT (to investigate the course of PPI therapy); (7) severe heart failure, severe hepatic or renal insufficiency, or severe immunological diseases; or (8) loss to follow-up or missing clinical data collected in this article.

### 2.2 Bleeding risk assessment

We used the 2015 European Cardiology Society (ESC) criteria (Roffi et al., 2016) to define patients at high risk of GIB: a history of GIB; chronic use of nonsteroidal anti-inflammatory drugs (NSAIDs); chronic use of glucocorticoids; anticoagulant therapy; or at least two of the following risk factors: age ≥65 years, dyspepsia, gastroesophageal reflux disease, and chronic alcohol use (*Helicobacter pylori* infection, an ESC risk factor, was not included because relevant information could not be obtained from the hospital information system (HIS)). However, in our study, patients with a history of progressive peptic ulcers or ulcer recurrence/bleeding within 3 months prior to admission, dyspepsia, gastroesophageal reflux disease, GIB, or anticoagulant therapy were excluded. Therefore, ultimately, patients with long-term use of NSAIDs, long-term use of glucocorticoids, or two risk factors (age ≥65 years and history of long-term alcohol use) were classified as having a high risk for GIB and excluded, whereas other patients were classified as having a low risk for GIB and considered eligible for this study.

### 2.3 Variables and definitions

Data were collected from the hospital information system (HIS) through postdischarge follow-up assessments. The following characteristics were recorded.

(1) Demographic information: Age, sex, smoking history, drinking history, etc.
(2) Laboratory test results: Glycated haemoglobin (HbA1c), platelet count (PLT), haemoglobin (Hb), haematocrit, and creatinine levels.
(3) Comorbidities: Conditions such as hypertension, diabetes, hyperlipidaemia, coronary artery disease, and history of liver disease.
(4) DAPT: Types of medications used and duration of treatment.
(5) PPI use: Prophylactic use of PPIs at the initiation of DAPT and the duration of PPI treatment. Since this study excluded patients with planned PPI use for treating acid-related disorders (such as gastroesophageal reflux disease, GIB, or persistent gastrointestinal discomfort), the use of PPIs concomitantly with DAPT initiation was defined as prophylactic PPI use, whereas nonprophylactic use was defined as no PPI use during this period (Fang et al., 2022). The OPT-PEACE study suggested that gastrointestinal mucosal erosions and ulcers progress rapidly within the first 3–7 days of DAPT initiation (Han et al., 2022). Therefore, we chose 7 days as the cut-off value for the PPI treatment course and divided courses into ≤7 days and >7 days for separate analyses.

All patients were followed up for 1 year, with standardized telephone interviews conducted by certified external clinical assessors. All interviews were recorded and traceable.

### 2.4 Clinical outcomes and definitions

The primary clinical outcome measures recorded within 1 year of follow-up were GIB and gastrointestinal discomfort. The secondary clinical outcomes included other types of bleeding and the occurrence of pneumonia.

The diagnosis of GIB was confirmed when a patient presented symptoms such as vomiting blood, black stools, or bloody stools, with or without accompanying signs of circulatory failure such as dizziness, palpitations, pallor, increased heart rate, and lowered blood pressure; this diagnosis is made primarily by clinical doctors (Chinese Journal of Internal Medicine et al., 2019). Gastrointestinal discomfort was defined as the presence of symptoms such as nausea, vomiting, belching, acid reflux, a retrosternal burning sensation, and upper abdominal pain, which required medical treatment or self-medication for relief. Other types of bleeding were defined as any bleeding event outside the gastrointestinal tract occurring during hospitalization or follow-up or a decrease in haemoglobin levels of at least 4 g/dL (Zhou et al., 2022). Pneumonia was diagnosed by clinicians during the DAPT period based on the patient’s symptoms, physical signs, and chest CT scan results.

### 2.5 Statistical analysis

Statistical analysis was conducted using SPSS 23.0 (IBM, Armonk, NY, USA). Categorical data are presented as frequencies and percentages and were compared using the χ^2^ test or Fisher’s exact test. Continuous data following a normal distribution are expressed as x ± s and were compared using one-way ANOVA. Nonparametric data were compared using the *Mann–Whitney U* test, with the significance level set at P < 0.05. Logistic regression analysis was used to evaluate the risk factors associated with the four outcome measures: GIB, gastrointestinal discomfort, other types of bleeding, and pneumonia. The analysis began with univariate logistic regression to preliminarily explore the correlations between confounding factors and different outcome measures by calculating P values, odds ratios (ORs) and 95% confidence intervals (95% CIs), particularly regarding the impact of PPI usage and treatment duration, to understand the factors potentially affecting the clinical outcomes of patients at low risk of GIB receiving DAPT. This was followed by multivariate logistic regression, incorporating variables with P < 0.20 from the univariate analysis along with the use of PPIs. A stepwise multivariate regression model was used to identify independent predictors specifically related to clinical outcomes, particularly the effects of PPI usage and treatment duration, in patients at low risk of GIB receiving DAPT. The strength of the association between independent risk factors and specific clinical outcomes was assessed by calculating adjusted odds ratios (adjusted ORs) and 95% confidence intervals (95% CIs). We used G*Power software to perform *post hoc* power analysis on the primary clinical outcomes of this study, GIB and gastrointestinal discomfort, to test the reliability of the research results. Power values ≥0.8 were considered high, indicating a high probability of detecting a truly existing effect. Power values ≥0.2 and <0.8 were considered moderate, indicating a certain ability to detect a truly existing effect. Power values <0.2 were considered low, indicating that even if a true effect exists, the probability that the study can detect this effect is very low (Faul et al., 2007).

## 3 Results

### 3.1 Clinical characteristics of the included patients

**FIGURE 1:**
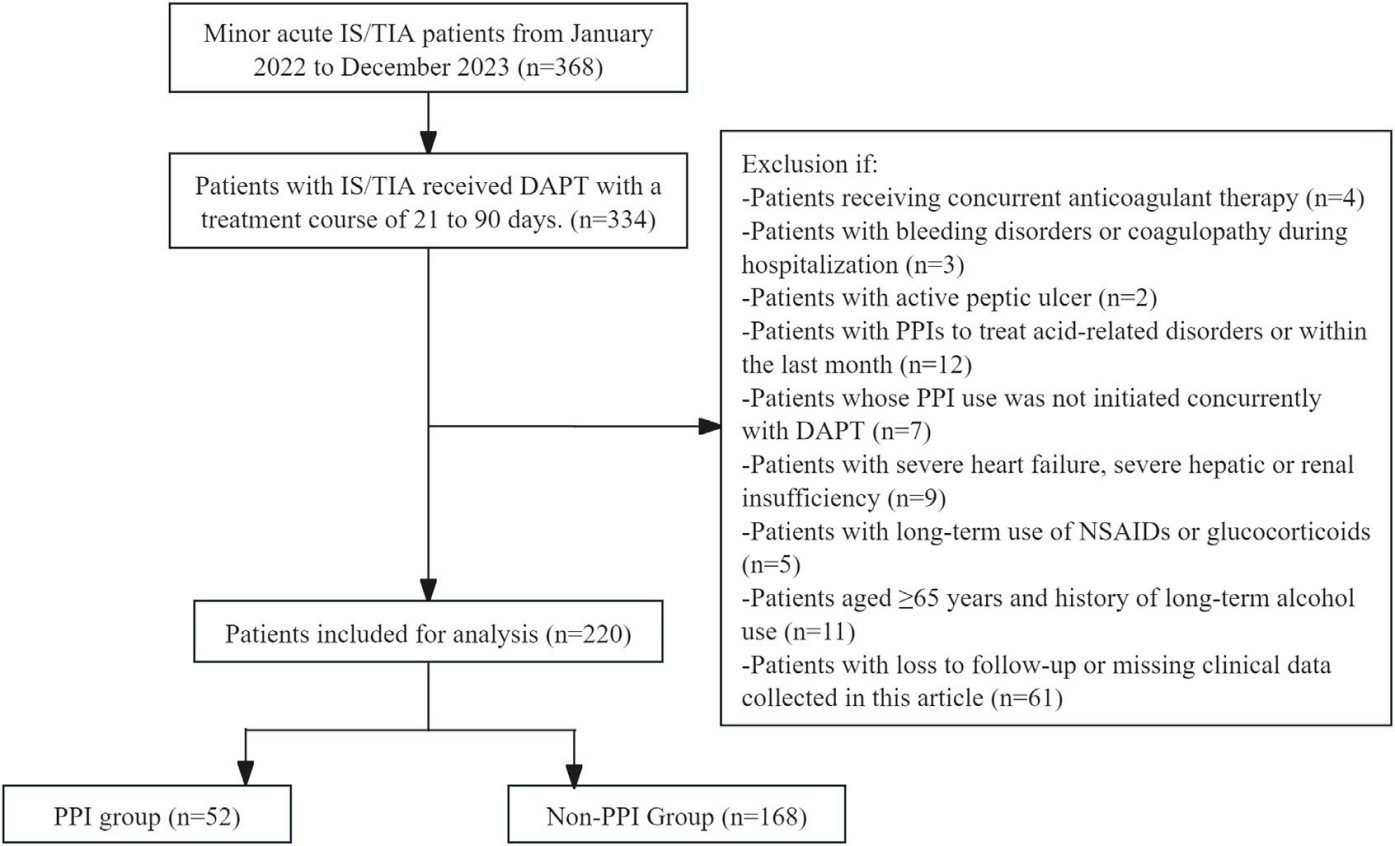
The flow chart of patient inclusion and screening.

This study included 220 patients with minor IS/TIA who were at low risk for GIB and who received dual-antiplatelet therapy (DAPT), as shown in Figure 1. Fifty-two patients (23.64%) used proton pump inhibitors (PPIs) in combination with their DAPT regimen. Among these patients, 39 were male (75.00%), with an average age of 64.25 ± 10.25 years. Twenty-six patients (50.00%) were older than 65. The majority of these patients (50 out of 52, 96.15%) were treated with a DAPT regimen consisting of aspirin combined with clopidogrel, whereas only two patients (3.85%) received a regimen of aspirin combined with ticagrelor. Thirty-two patients (61.54%) had a DAPT duration of 21 days, and 20 patients (38.46%) had a duration between 22 and 90 days. There were 3 instances of GIB (5.77%), 10 instances of gastrointestinal discomfort symptoms (19.23%), 3 instances of other types of bleeding (5.77%)—primarily gingival bleeding and skin bruising—and 3 cases of pneumonia (5.77%) in the PPI group. Among the 168 patients (76.36%) who did not use PPIs during DAPT, 119 were male (70.83%), with an average age of 62.39 ± 11.10 years. Seventy-one patients (42.26%) were older than 65. Similar to the PPI group, the majority (162 out of 168, 96.43%) received DAPT with aspirin combined with clopidogrel, whereas only 6 (3.57%) received aspirin combined with ticagrelor. Ninety-seven patients (57.74%) had a DAPT duration of 21 days, and 71 patients (42.26%) had a duration between 22 and 90 days. There were 2 cases of GIB (1.20%), 60 cases of gastrointestinal discomfort (35.71%), 13 cases of other types of bleeding (7.74%)—primarily gingival bleeding and skin bruising—and 4 cases of pneumonia (2.38%) in the non-PPI group. There were no statistically significant differences in the remaining characteristics between the two groups (*P* > 0.05). The specific details are provided in Table 1.

**TABLE 1:** Clinical characteristics of included patients.

Variables	PPI Group (n = 52)	Non-PPI Group (n = 168)	x^2^/Z/t	*P*-value
Male (n, %)	39 (75.00)	119 (70.83)	0.341	0.559
Age (years, $\bar{x}$ ± s)	64.25 ± 10.25	62.39 ± 11.10	−1.073	0.285
Age ≥65 years (n, %)	26 (50.00)	71 (42.26)	0.965	0.326
Smoking history (n, %)	11 (21.15)	39 (23.21)	0.096	0.757
Drinking history (n, %)	6 (11.54)	24 (14.29)	0.254	0.614
Glycated hemoglobin (%, $\bar{x}$ ± s)	6.50 ± 1.21	6.25 ± 1.31	−1.215	0.226
Platelet count (×10^9^/L, $\bar{x}$ ± s)	220.08 ± 77.05	210.56 ± 54.07	−0.993	0.322
Hemoglobin (g/L, $\bar{x}$ ± s)	138.75 ± 13.90	137.52 ± 15.34	−0.514	0.608
Hematocrit (%, $\bar{x}$ ± s)	0.42 ± 0.04	0.42 ± 0.04	−0.273	0.785
Creatinine (umol/L, $\bar{x}$ ± s)	63.71 ± 21.47	64.09 ± 16.77	−0.131	0.896
Hypertension (n, %)	33 (63.46)	108 (64.29)	0.012	0.914
Diabetes (n, %)	16 (30.77)	43 (25.60)	0.542	0.462
Hyperlipidemia (n, %)	17 (32.69)	40 (23.81)	1.632	0.201
Coronary heart disease (n, %)	7 (13.46)	24 (14.29)	0.022	0.881
History of liver disease (n, %)	2 (3.85)	4 (2.38)	0.006	0.936
Antiplatelet regimen (n, %)			0.009	0.926
Aspirin + Clopidogrel	50 (96.15)	162 (96.43)		
Aspirin + Ticagrelor	2 (3.85)	6 (3.57)		
Duration of DAPT (n, %)			0.236	0.627
21 days	32 (61.54)	97 (57.74)		
22∼90 days	20 (38.46)	71 (42.26)		
Clinical outcomes (n, %)				
Gastrointestinal bleeding	3 (5.77)	2 (1.20)	1.970	0.160
Gastrointestinal discomfort	10 (19.23)	60 (35.71)	4.973	0.026
Other types of bleeding	3 (5.77)	13 (7.74)	0.030	0.863
Pneumonia	3 (5.77)	4 (2.38)	0.584	0.445

### 3.2 Association between PPI use and the occurrence of GIB

Among the 220 patients with minor IS/TIA at low risk for GIB receiving short-term DAPT, only 5 patients experienced GIB. Univariate logistic analysis indicated that the combined use of PPIs, the type of short-term DAPT, and the duration of DAPT were not related to the occurrence of GIB. Multivariate logistic analysis identified a history of smoking as an independent risk factor for GIB in these patients (60.00% vs. 21.86%, OR: 7.143; 95% CI: 1.030–50.000). However, the *post hoc* power value was 28%, indicating that the statistical analysis was limited by the small sample size of patients with GIB (Table 2).

**TABLE 2:** Univariate and multivariate logistic analysis of GIB and gastrointestinal discomfort.

Variable	Univariate analysis			Multivariate analysis		Univariate analysis			Multivariate analysis	
	With GIB (n = 5)	Without GIB (n = 215)	P-value	P-value	OR (95%CI)	With gastrointestinal discomfort (n = 70)	Without gastrointestinal discomfort (n = 150)	P-value	P-value	OR (95%CI)
Male (n, %)	4 (80.00%)	154 (71.63%)	0.691	—	—	49 (70.00%)	109 (72.67%)	0.682	—	—
Age (years, $\bar{x}$ ± s)	66.40 ± 8.76	62.75 ± 10.96	0.391	—	—	63.11 ± 11.06	62.70 ± 10.88	0.793	—	—
Age ≥65 years (n, %)	3 (60.00%)	94 (43.72%)	0.419	—	—	30 (42.85%)	67 (44.67%)	0.801	-	—
Smoking history (n, %)	3 (60.00%)	47 (21.86%)	0.081	0.047	7.143 (1.030–50.000)	16 (22.86%)	34 (22.67%)	0.975	—	—
Drinking history (n, %)	0 (0.00%)	30 (13.95%)	0.998	—	—	8 (11.43%)	22 (14.67%)	0.516	—	—
Glycated hemoglobin (%, $\bar{x}$ ± s)	7.34 ± 1.89	6.29 ± 1.27	0.106	0.416	0.742 (0.361–1.524)	6.47 ± 1.25	6.24 ± 1.30	0.226	—	—
Platelet count (×10^9^/L, $\bar{x}$ ± s)	215.80 ± 48.81	212.76 ± 60.63	0.970	—	—	201.23 ± 51.21	218.20 ± 63.51	0.055	0.064	0.995 (0.990–1.000)
Hemoglobin (g/L, $\bar{x}$ ± s)	142.80 ± 12.93	137.70 ± 15.04	0.481	—	—	137.60 ± 13.88	137.92 ± 15.52	0.881	—	—
Hematocrit (%, $\bar{x}$ ± s)	0.43 ± 0.04	0.42 ± 0.04	0.460	—	—	0.42 ± 0.040	0.41 ± 0.04	0.834	—	—
Creatinine (umol/L, $\bar{x}$ ± s)	65.27 ± 11.49	63.97 ± 18.08	0.840	—	—	64.06 ± 16.82	63.97 ± 18.50	0.971	—	—
Hypertension (n, %)	2 (40.00%)	139 (64.65%)	0.260	—	—	48 (68.57%)	93 (62.00%)	0.345	—	—
Diabetes (n, %)	3 (60.00%)	56 (26.05%)	0.128	0.102	4.926 (0.727–33.333)	21 (30.00%)	38 (25.33%)	0.467	—	—
Hyperlipidemia (n, %)	1 (20.00%)	56 (26.05%)	0.705	—	—	14 (20.00%)	43 (28.67%)	0.174	0.218	1.572 (0.766–3.218)
CHD (n, %)	1 (20.00%)	30 (13.95%)	0.697	—	—	9 (12.86%)	22 (14.67%)	0.720	—	—
History of liver disease (n, %)	0 (0.00%)	6 (2.79%)	0.999	—	—	4 (5.71%)	2 (1.33%)	0.088	0.032	6.211 (1.168–33.333)
Combined use of PPI (n, %)	3 (60.00%)	49 (22.79%)	0.081	0.059	6.410 (0.930–43.478)	10 (14.29%)	42 (28.00%)	0.029	0.033	0.448 (0.215–0.935)
Antiplatelet regimen (n, %)			0.998	—	—			0.068	0.036	2.793 (1.068–7.299)
Aspirin + Clopidogrel	5 (100.00%)	207 (96.28%)	-	-	—	66 (94.29%)	146 (97.33%)	—	—	—
Aspirin + Ticagrelor	0 (0.00%)	8 (3.72%)	-	-	—	4 (5.71%)	4 (2.67%)	—	—	—
Duration of DAPT (n, %)			0.419	-	—			0.523	-	-
21 days	2 (40.00%)	127 (59.07%)	—	—	—	37 (52.86%)	92 (61.33%)	—	—	—
22∼90 days	3 (60.00%)	88 (40.93%)	—	—	—	33 (47.14%)	58 (38.67%)	—	—	—

### 3.3 Association between PPI use and gastrointestinal discomfort

Seventy patients experienced symptoms of gastrointestinal discomfort, with a *post hoc* power of 71%, which was a moderately high power, indicating that the research results reflected the true existence of an effect to a certain extent. Univariate logistic analysis revealed that the combined use of PPIs during short-term DAPT significantly reduced the occurrence of gastrointestinal discomfort (P = 0.029). Multivariate logistic analysis revealed that a history of liver disease (5.71% vs. 1.33%, OR: 6.211; 95% CI: 1.168–33.333) and the use of aspirin combined with ticagrelor (5.71% vs. 2.67%, OR: 2.793; 95% CI: 1.068–7.299) were independent risk factors for gastrointestinal discomfort. The combined use of PPIs was an independent protective factor against gastrointestinal discomfort (14.29% vs. 28.00%, OR: 0.448; 95% CI: 0.215–0.935) (Table 2).

### 3.4 Association between PPI use and other types of bleeding

Sixteen patients experienced other types of bleeding, such as gum bleeding and skin bruising. Univariate logistic analysis suggested that age and type of short-term DAPT might be related to the occurrence of these other types of bleeding (P = 0.014, P = 0.018, P = 0.005). Multivariate logistic analysis revealed that the combination of aspirin and ticagrelor was an independent risk factor for other types of bleeding (12.50% vs. 2.94%, OR: 6.135; 95% CI: 1.724–21.277). The use of PPIs was not related to the occurrence of other types of bleeding in these patients (Table 3).

**TABLE 3:** Univariate and multivariate logistic analysis of other types of bleeding and pneumonia.

Variable	Univariate analysis			Multivariate analysis		Univariate analysis			Multivariate analysis	
	With other types of bleeding (n = 16)	Without other types of bleeding (n = 204)	P-value	P-value	Or (95% CI)	With pneumonia (n = 7)	Without pneumonia (n = 213)	P-value	P-value	Or (95% CI)
Male (n, %)	11 (68.75%)	147 (72.06%)	0.466	—	—	4 (57.14%)	154 (72.30%)	0.715	-	—
Age (years, $\bar{x}$ ± s)	58.19 ± 8.80	63.20 ± 11.00	0.014	0.426	1.033 (0.953–1.120)	54.71 ± 14.73	63.10 ± 10.71	0.040	0.143	0.966 (0.923–1.012)
Age ≥65 years (n, %)	3 (18.75%)	94 (46.08%)	0.018	0.083	6.587 (0.782–55.479)	2 (28.57%)	95 (44.60%)	0.238	-	—
Smoking history (n, %)	3 (18.75%)	47 (23.04%)	0.889	—	—	0 (0.00%)	50 (23.47%)	0.997	-	—
Drinking history (n, %)	1 (6.25%)	29 (14.22%)	0.267	—	—	1 (14.29%)	29 (13.62%)	0.827	-	—
Glycated hemoglobin (%, $\bar{x}$ ± s)	5.92 ± 0.59	6.34 ± 1.33	0.124	0.130	0.616 (0.329–1.154)	6.56 ± 1.43	6.30 ± 1.29	0.973	-	—
Platelet count (×10^9^/L, $\bar{x}$ ± s)	229.47 ± 36.06	211.60 ± 61.59	0.216	—	—	194.86 ± 88.22	213.42 ± 59.32	0.476	-	—
Hemoglobin (g/L, $\bar{x}$ ± s)	142.27 ± 15.59	137.49 ± 14.92	0.193	0.112	0.897 (0.785–1.626)	129.00 ± 18.66	138.11 ± 14.81	0.303	-	—
Hematocrit (%, $\bar{x}$ ± s)	0.44 ± 0.04	0.42 ± 0.04	0.299	—	—	0.40 ± 0.04	0.42 ± 0.042	0.410	-	—
Creatinine (umol/L, $\bar{x}$ ± s)	60.04 ± 13.17	64.31 ± 18.25	0.188	0.892	1.003 (0.966–1.041)	73.67 ± 45.97	63.68 ± 16.42	0.283	-	—
Hypertension (n, %)	12 (75.00%)	129 (63.24%)	0.147	0.231	2.268 (0.594–8.621)	5 (71.43%)	136 (63.85%)	0.415	-	—
Diabetes (n, %)	3 (18.75%)	56 (27.45%)	0.200	0.591	0.608 (0.134–2.751)	3 (42.86%)	56 (26.29%)	0.693	-	—
Hyperlipidemia (n, %)	2 (12.50%)	55 (26.96%)	0.433	—	—	3 (42.86%)	54 (25.35%)	0.070	0.141	3.352 (0.669–16.803)
CHD (n, %)	0 (0.00%)	31 (15.20%)	0.998	—	—	1 (14.29%)	30 (14.08%)	0.801	-	—
History of liver disease (n, %)	0 (0.00%)	6 (2.94%)	0.999	—	—	0 (0.00%)	6 (2.82%)	0.999	-	—
Combined use of PPI (n, %)	3 (18.75%)	49 (24.02%)	0.889	0.916	0.926 (0.223–3.851)	3 (42.86%)	49 (23.00%)	0.494	0.840	1.146 (0.304–4.322)
Antiplatelet regimen (n, %)			0.005	0.004	6.135 (1.724–21.277)			0.032	0.014	7.042 (1.475–33.333)
Aspirin + Clopidogrel	14 (87.50%)	198 (97.06%)	—	—	—	6 (85.71%)	206 (96.71%)	—	—	—
Aspirin + Ticagrelor	2 (12.50%)	6 (2.94%)	—	—	—	1 (14.29%)	7 (3.29%)	—	—	—
Duration of DAPT (n, %)			0.221	-	—			0.877	-	—
21 days	7 (43.75%)	122 (59.80%)	—	—	—	5 (71.43%)	124 (58.22%)	—	—	—
22∼90 days	9 (56.25%)	82 (40.20%)	—	—	—	2 (28.57%)	89 (41.78%)	—	—	—

### 3.5 Association between PPI use and pneumonia

Among the 220 patients who received short-term DAPT, 7 developed pneumonia, and the small sample size limited the power of the statistical analysis to a certain extent. Univariate logistic analysis indicated that age and type of short-term DAPT might be related to the occurrence of pneumonia (P = 0.040, P = 0.032). Multivariate logistic analysis revealed that the combination of aspirin and ticagrelor was an independent risk factor for pneumonia in these patients (14.29% vs. 3.29%, OR: 7.042; 95% CI: 1.475–33.333). The incidence of pneumonia in the PPI group was higher than that in the non-PPI group, but the difference was not statistically significant (42.86% vs. 23.00%, P = 0.840, OR: 1.146; 95% CI: 0.304–4.322) (Table 3).

### 3.6 Association between PPI duration and incidence of gastrointestinal discomfort

#### 3.6.1 Clinical characteristics

This study included 52 patients with a low risk of GIB and minor IS/TIA who received DAPT in conjunction with PPIs. Most patients (40 patients, 76.92%) were on a PPI regimen of less than 7 days, including 32 males (80.00%), with an average age of 63.78 ± 10.54 years; 19 patients (47.50%) were older than 65. The majority (39 patients, 97.50%) were treated with a DAPT regimen consisting of aspirin combined with clopidogrel, whereas one patient (2.50%) was treated with a regimen of aspirin combined with ticagrelor. Twenty-four patients (60.00%) had a DAPT duration of 21 days, with the remainder on a regimen lasting 22–90 days. A smaller group (12 patients, 23.08%) was on a PPI regimen of more than 7 days, with the longest duration being 90 days. This group included 7 males (58.33%), with an average age of 65.83 ± 9.48 years; 7 patients (58.33%) were older than 65. Most of these patients (11, 91.67%) used a DAPT regimen of aspirin combined with clopidogrel, and one patient (8.33%) used a regimen of aspirin combined with ticagrelor. Eight of these patients (66.67%) had a DAPT duration of 21 days, with the remaining patients having durations of 22–90 days (Table 4).

**TABLE 4:** Comparison of clinical characteristics between patients with different PPI durations.

Variable	PPI ≤7 days (n = 40)	PPI >7 days (n = 12)	x^2^/Z/t	P-value
Age (years, $\bar{x}$ ± s)	63.78 ± 10.54	65.83 ± 9.48	−0.606	0.547
Age ≥65 years (n, %)	19 (47.50%)	7 (58.33%)	0.433	0.510
Smoking history (n, %)	10 (25.00%)	1 (8.33%)	0.700	0.403
Drinking history (n, %)	3 (7.50%)	3 (25.00%)	1.320	0.251
Glycated hemoglobin (%, $\bar{x}$ ± s)	6.49 ± 1.09	6.55 ± 1.61	−0.155	0.877
Platelet Count (×10^9^/L, $\bar{x}$ ± s)	227.73 ± 80.32	194.58 ± 61.07	1.316	0.194
Hemoglobin (g/L, $\bar{x}$ ± s)	139.43 ± 14.51	136.50 ± 11.89	0.636	0.528
Hematocrit (%, $\bar{x}$ ± s)	0.42 ± 0.04	0.41 ± 0.04	0.714	0.479
Creatinine (umol/L, $\bar{x}$ ± s)	67.32 ± 22.24	61.68 ± 13.39	2.305	0.125
Hypertension (n, %)	26 (65.00%)	7 (58.33%)	0.177	0.674
Diabetes (n, %)	12 (30.00%)	4 (33.33%)	0.048	0.826
Hyperlipidemia (n, %)	11 (27.50%)	6 (50.00%)	2.124	0.145
CHD (n, %)	5 (12.50%)	2 (16.67%)	0.000	1.000
History of liver disease (n, %)	2 (5.00%)	0 (0.00%)	0.624	0.430
Antiplatelet regimen (n, %)			0.004	0.948
Aspirin + Clopidogrel	39 (97.50%)	11 (91.67%)		
Aspirin + Ticagrelor	1 (2.50%)	1 (8.33%)		
DAPT Duration (n, %)			0.173	0.677
21 days	24 (60.00%)	8 (66.67%)		
22∼90 days	16 (40.00%)	4 (33.33%)		
Clinical outcomes (n, %)				
GIB	3 (7.50%)	0 (0.00%)	0.955	0.328
Other types of bleeding	2 (5.00%)	1 (8.33%)	0.000	1.000
Gastrointestinal discomfort	7 (17.50%)	3 (25.00%)	0.026	0.872
Pneumonia	1 (2.50%)	2 (16.67%)	1.300	0.254

#### 3.6.2 Association between PPI duration (≤7 days and >7 days) and gastrointestinal discomfort

Among the patients at low risk of GIB who underwent short-term DAPT with concomitant PPI treatment, 10 experienced symptoms of gastrointestinal discomfort. Both univariate and multivariate logistic regression analyses indicated that the incidence of gastrointestinal discomfort was similar between those who were on PPIs for ≤7 days and those who were on PPIs for >7 days. These findings suggest that extending the PPI regimen beyond 7 days does not reduce the incidence of gastrointestinal discomfort in patients with minor IS or TIA receiving short-term DAPT. These findings are detailed in Table 5.

**TABLE 5:** Univariate and multivariate logistic analysis of gastrointestinal discomfort in patients with different PPI durations.

Variable	Univariate analysis				Multivariate analysis	
	With GI discomfort (n = 10)	Without GI discomfort (n = 42)	P-value	Or (95% CI)	P-value	Or (95% CI)
Male (n, %)	8 (80.00%)	31 (73.81%)	0.686	1.419 (0.261–7.733)	—	—
Age (years, $\bar{x}$ ± s)	60.90 ± 10.15	65.05 ± 10.23	0.252	0.961 (0.897–1.029)	—	—
Age ≥65 years (n, %)	4 (40.00%)	22 (52.38%)	0.484	1.650 (0.406–6.709)	—	—
Smoking history (n, %)	2 (20.00%)	9 (21.43%)	0.921	1.091 (0.196–6.067)	—	—
Drinking history (n, %)	1 (10.00%)	5 (11.90%)	0.866	1.216 (0.126–11.740)	—	—
Glycated hemoglobin (%, $\bar{x}$ ± s)	6.38 ± 0.72	6.53 ± 1.31	0.721	0.895 (0.486–1.648)	—	—
Platelet Count (×10^9^/L, $\bar{x}$ ± s)	209.00 ± 46.54	222.71 ± 82.90	0.611	0.997 (0.987–1.007)	—	—
Hemoglobin (g/L, $\bar{x}$ ± s)	142.50 ± 13.04	137.86 ± 14.09	0.344	1.027 (0.972–1.084)	—	—
Hematocrit (%, $\bar{x}$ ± s)	0.43 ± 0.04	0.42 ± 0.04	0.280	$\infty \left(0-\infty \right)$	—	—
Creatinine (umol/L, $\bar{x}$ ± s)	62.01 ± 13.99	64.12 ± 23.02	0.779	0.995 (0.960–1.031)	—	—
Hypertension (n, %)	7 (70.00%)	26 (61.90%)	0.634	0.696 (0.157–3.087)	—	—
Diabetes (n, %)	1 (10.00%)	15 (35.71%)	0.144	5.000 (0.576–43.366)	0.139	0.195 (0.022–1.704)
Hyperlipidemia (n, %)	3 (30.00%)	14 (33.33%)	0.840	1.167 (0.261–5.213)	—	—
CHD (n, %)	1 (10.00%)	6 (14.29%)	0.723	1.500 (0.160–14.083)	—	—
History of liver disease (n, %)	0 (0.00%)	2 (4.76%)	0.999	$\infty \left(0-\infty \right)$	—	—
PPI >7 days (n, %)	3 (30.00%)	9 (21.43%)	0.565	0.636 (0.136–2.969)	0.520	1.686 (0.343–8.333)
Antiplatelet Regimen (n, %)			0.299	0.220 (0.013–3.849)	—	—
Aspirin + Clopidogrel	9 (90.00%)	41 (97.62%)				
Aspirin + Ticagrelor	1 (10.00%)	1 (2.38%)				
DAPT Duration (n, %)			0.543	1.587 (0.359–7.014)	—	—
21 days	7 (70.00%)	25 (59.52%)				
22∼90 days	3 (30.00%)	17 (40.48%)				

## 4 Discussion

Stroke is the third leading cause of death in China and the primary cause of disability-adjusted life years (DALYs). According to the 2019 Global Burden of Disease (GBD) study, there were 2.87 million new cases of IS and 24.18 million existing cases in China in 2019. The number of DALYs associated with IS has reached 45.9 million, representing a 36.7% increase since 1990 (Wang et al., 2023), placing a significant burden on healthcare systems both in China and globally. DAPT has been widely recommended by multiple international guidelines as the core strategy for the treatment and secondary prevention of IS and TIA (Valgimigli et al., 2017; National Clinical Guideline for Stroke, 2023; Kleindorfer et al., 2021). However, the gastrointestinal injury associated with DAPT, particularly GIB, is a critical concern. A recent study revealed that the mortality rate of patients taking oral antiplatelet drugs who experienced GIB was approximately 20% higher than that of patients without GIB (Lv et al., 2020). Gastrointestinal injuries induced by DAPT (including severe complications and gastrointestinal symptoms) reach a peak in the first 3 months posttreatment (Zhong et al., 2018).

There are many antiplatelet drugs available on the market, and their bleeding tendencies vary. Among the most commonly used antiplatelet drugs in clinical practice, aspirin and clopidogrel, when used for secondary prevention of IS/TIA, the incidence of bleeding in clopidogrel was significantly lower (6.1% vs. 4.5%), especially safer in populations at high risk of bleeding (Kang et al., 2025). Regarding the bleeding risk of relatively newer antiplatelet drugs, the research results of Wang Yong et al. showed that in patients with non-ST-segment elevation ACS undergoing PCI, the overall incidence of bleeding events in the ticagrelor group was significantly higher than that in the clopidogrel group (6.7% vs. 4.7%). Among them, the incidence of minor bleeding events was higher in the ticagrelor group than in the clopidogrel group (4.5% vs. 2.8%), while the incidence of major bleeding events was similar between the two groups (P > 0.05) (Li et al., 2023). The results of the TRITON-TIMI-38 clinical trial showed that in ACS patients undergoing PCI, compared with clopidogrel, prasugrel had a higher incidence of non-fatal bleeding (1.1% vs. 0.9%) and more common fatal bleeding (0.4% vs. 0.1%) (Wiviott et al., 2007). A meta-analysis showed that severe or life-threatening bleeding was similar between cangrelor and clopidogrel (pooled OR 1.21, 95% CI: 0.70–2.12, p = 0.50, I^2^ = 0%) (Pandit et al., 2014). The INSURE clinical trial published in 2023 mainly compared the efficacy and safety of indobufen and aspirin in patients with acute moderate to severe ischemic stroke. The results showed that there were 18 cases (0.7%) of moderate to severe bleeding in the indobufen group and 28 cases (1.0%) in the aspirin group (0.63, 95% CI 0.35–1.15; P = 0.13), indicating that the risk of moderate to severe bleeding caused by the two was basically the same (Pan et al., 2023). A meta-analysis published in 2019, which included 10 studies, showed that cilostazol monotherapy had a lower risk of hemorrhagic stroke than traditional single antiplatelet therapy (mainly aspirin) (Kim et al., 2019).

PPIs are the first-line agents for preventing DAPT-related GI injury. In patients at high risk of GIB receiving DAPT after IS/TIA, guidelines from the UK, the USA, Ireland, and China consistently recommend the use of PPIs for GI protection (Expert Committee on the Prevention and Treatment Guidelines for Thrombotic Diseases in China Chinese, 2018; National Clinical Guideline for Stroke, 2023; Saven et al., 2022). However, for patients at low risk of GIB, the recommendations are inconsistent. The American guidelines adopt a conservative approach, advising against routine PPI use, whereas other guidelines continue to endorse it (Expert Committee on the Prevention and Treatment Guidelines for Thrombotic Diseases in China Chinese, 2018; National Clinical Guideline for Stroke, 2023; Saven et al., 2022). Moreover, clinical research exploring the benefits and risks of PPI use in this population remains scarce, leading to substantial uncertainty among clinicians regarding whether to prescribe PPIs and the optimal duration of use in minor IS/TIA patients at low risk of GIB undergoing short-term DAPT. Additionally, studies have shown that PPIs are often overprescribed without clear indications, with consumption increasing annually in both Western and Eastern countries. Mohammad et al. reported that over 86% of general ward patients used PPIs inappropriately (Alqudah et al., 2016). Given these concerns, we conducted this study.

Our study explored the current status of prophylactic PPI use, the benefits of this practice in preventing gastrointestinal damage, and the recommended course of treatment in minor IS/TIA patients at low risk of GIB receiving short-term DAPT. The results revealed that the rate of prophylactic PPI use in such patients in our hospital was less than 1/4, which was lower than the 27%–71% reported in previous studies. This may be related to the fact that this study included only patients at low risk of GIB, and there are no clear guidelines recommending PPI use in patients at low risk of GIB, leading clinicians to prescribe PPIs on the basis of their clinical experience and medication habits. Additionally, the concerns of doctors regarding adverse reactions and drug interactions associated with long-term PPI use, including the reduced efficacy of DAPT due to interactions with antiplatelet drugs such as clopidogrel, as well as increased risks of chronic kidney disease, cardiovascular events, and community-acquired pneumonia, might partially explain this result (Lazarus et al., 2016; Northuis et al., 2023; Othman et al., 2016; Sehested et al., 2018).

In this study, a total of 5 patients developed GIB during follow-up, with an incidence rate of 2.3%, which was consistent with reports in the literature (2.3%∼7%) (Li et al., 2017). The results revealed that there was no significant difference between prophylactic use and nonuse of PPIs in reducing the incidence of GIB in minor IS/TIA patients at low risk of GIB receiving short-term DAPT. This finding was inconsistent with some recently published studies and our research expectation that PPI use could reduce the incidence of gastrointestinal bleeding in such patients (Sehested et al., 2019; Baik et al., 2025). However, considering the small number of patients with GIB and the *post hoc* power analysis showing a power of only 28%, we believe that this result was due mainly to the low incidence of events. The findings of some previous studies were consistent with our conclusions. For example, a multicentre study by Zhou et al., in 2021, which included 25,567 ACS patients receiving DAPT, revealed that PPIs could not effectively prevent all types of GIB in the acute phase, especially types of lower gastrointestinal bleeding such as bleeding in the small intestine (Zhou et al., 2022).

Our study demonstrated that prophylactic PPI use was an independent protective factor against GI discomfort, reducing its incidence by approximately twofold. This finding aligns with the pharmacological mechanisms of PPIs. DAPT, especially aspirin, inhibits cyclooxygenase (COX) activity and directly damages the gastrointestinal mucosa, leading to gastrointestinal bleeding, ulcers and other complications, whereas PPIs modify the thiol groups of the H + -K + -ATPase enzyme, inhibiting its activity and blocking the final step of gastric acid secretion. This action suppresses basal and stimulated acid secretion induced by histamine, acetylcholine, gastrin, and food intake, thereby protecting the gastric mucosa and mitigating DAPT-induced GI injury (Zhou et al., 2022; Zhu et al., 2022).

With respect to other types of bleeding, PPI use showed no significant difference, which is consistent with the findings of previous studies (Pan et al., 2021; Zhou et al., 2022). A recent Danish nationwide registry-based study involving 46,301 post-myocardial infarction patients on DAPT revealed that PPI use was significantly associated with a reduced risk of upper GIB (OR: 0.62; 95% CI: 0.48–0.77) but was not associated with all-cause bleeding (OR: 0.88; 95% CI: 0.74–1.05) or lower GIB (OR: 1.06; 95% CI: 0.82–1.33) (Sehested et al., 2019). In our study, patients receiving PPIs had a 1.2-fold greater incidence of pneumonia than did those not receiving PPIs, although the difference was not statistically significant. A population-based Swedish nationwide study with a self-controlled case series design conducted between 2005 and 2019 revealed that among 519,152 patients with at least one episode of pneumonia during the study period, 307,709 PPI treatment cycles occurred. After PPI use, the overall risk of pneumonia increased by 73% (IRR: 1.73, 95% CI: 1.71–1.75) (Maret-Ouda et al., 2023). Another study conducted in the UK with 48,451 patients using a self-controlled design reported a slight increase in the risk of pneumonia during PPI use (IRR 1.19, 95% CI 1.14–1.25) (Othman et al., 2016). A meta-analysis published in 2020, including 7 studies and 65,590 pneumonia patients, revealed that PPI users had a 66% increased risk of pneumonia (OR: 1.66, 95% CI: 1.22–2.25) (Nguyen et al., 2020). Confusion regarding the indications for PPIs is a particular concern because gastroesophageal reflux disease (the main indication for PPI treatment) itself may increase the risk of pneumonia (Maret-Ouda et al., 2023). A large cohort study conducted in Canada, the UK, and the U.S. in 2014 evaluated 4,238,504 patients using NSAIDs with PPIs for ulcer prevention, attempting to avoid the influence of gastroesophageal reflux disease. The results revealed no correlation between PPI use and pneumonia (OR: 1.05, 95% CI: 0.89–1.25) (Filion et al., 2014). An RCT including 17,598 stable cardiovascular disease patients who started aspirin and/or rivaroxaban treatment and were randomly assigned to receive PPIs or placebo revealed that compared with placebo users, PPI users had no increased risk of pneumonia (IRR: 1.02, 95% CI: 0.87–1.19) (Moayyedi et al., 2019). A 2020 review of the available literature on this topic revealed that the role of PPI use in the aetiology of pneumonia remained unclear (Maret-Ouda et al., 2020). This study excluded the planned use of PPIs to treat acid-related disorders (such as gastroesophageal reflux disease, GIB, or persistent gastrointestinal discomfort), which to some extent supports the use of PPIs as a risk factor for pneumonia.

Based on these findings, we further compared the effectiveness of PPI treatment for ≤7 days versus >7 days in alleviating GI discomfort in minor IS/TIA patients at low risk of GIB undergoing short-term DAPT. No significant differences were observed, indicating that extending PPI therapy beyond 7 days does not enhance its efficacy. The 2021 OPT-PEACE study suggested that GI mucosal erosion and ulceration progress rapidly within the first 3–7 days of DAPT initiation, with some patients already experiencing occult bleeding or mild symptoms due to the mucosal healing cycle. Normal mucosal healing takes 72–94 h, but DAPT disrupts this process, leading to cumulative injury (Han et al., 2022). Early PPI coadministration within the first 7 days of DAPT initiation effectively minimizes mucosal damage and reduces GI discomfort.

This study has several advantages. First, most of the current relevant studies have focused on patients requiring long-term oral DAPT after ACS or undergoing PCI, whereas this study is the first to explore the prophylactic use of PPIs in reducing GI injury in this group of patients by using patients with minor IS/TIA who received short-term DAPT as the study population. Second, we included only IS/TIA patients with low GIB risk, focusing on this population to further clarify the necessity of the combined use of PPIs and to compensate for the shortcomings of the current guideline recommendations. Furthermore, this study is the first to analyse the duration of PPI use in this population, to clarify the shortest duration of treatment that can play an optimal role and to ensure clinical efficacy while avoiding the overuse of PPIs in the clinic. Finally, this study included several clinical outcomes, including gastrointestinal bleeding, gastrointestinal discomfort, other types of bleeding, and pneumonia, to explore the efficacy and safety of the combined use of PPIs in a multifaceted manner.

This study also has several limitations. (1) This was a retrospective study, with some differences in the homogeneity of the data and some missing data, which may have led to some bias in the study results. (2) The included data were from a single centre, and the results are influenced by the diagnosis and treatment level of physicians, which could lead to limitations in extrapolation of the results. (3) The number of patients included in this study was limited, and the number of patients with four clinical outcome indicators was small, which limits the power of the statistical analysis in detecting between-group differences to a certain extent and may introduce some bias to the research results. In the future, we will continue to supplement and analyse the relevant data. (4) *H. pylori* infection, a risk factor identified by the ESC, was not included because relevant information could not be obtained from the HIS. Additionally, the influence of dietary factors was not considered. All these factors may have caused a certain degree of bias in our research results.

## 5 Conclusion

In conclusion, the proportion of minor IS/TIA patients at low risk of GIB undergoing short-term DAPT and treatment with PPIs in China was low. PPI use significantly reduced GI discomfort, with similar efficacy between ≤7 days and >7 days of treatment. Although not statistically significant, a higher pneumonia rate was observed in the PPI group. Therefore, we recommend that minor IS/TIA patients at low risk of GIB who are receiving short-term DAPT should take PPIs regularly within the first 7 days of initiating DAPT, after which PPIs can be discontinued to reduce GI discomfort while minimizing adverse effects due to the overuse of PPIs. Of course, more high-quality studies with randomized, controlled, and multicentre designs and large sample sizes are still needed to verify the efficacy and safety of these results.

## Data Availability

The original contributions presented in the study are included in the article/supplementary material, further inquiries can be directed to the corresponding authors.
